# The Surveillance Outbreak Response Management and Analysis System (SORMAS): Digital Health Global Goods Maturity Assessment

**DOI:** 10.2196/15860

**Published:** 2020-04-29

**Authors:** Daniel Tom-Aba, Bernard Chawo Silenou, Juliane Doerrbecker, Carl Fourie, Carl Leitner, Martin Wahnschaffe, Maté Strysewske, Chinedu Chukwujekwu Arinze, Gerard Krause

**Affiliations:** 1 Helmholtz Centre for Infection Research Department of Epidemiology Braunschweig Germany; 2 Hannover Medical School (MHH) Hannover Germany; 3 Digital Square, PATH Chapel Hill, North Carolina North Carolina, NC United States; 4 Symeda GmbH Braunschweig Germany; 5 Nigeria Center for Disease Control Abuja Nigeria

**Keywords:** mHealth, eHealth, contact tracing, case management, epidemiology, Ebola Virus Disease, West Africa, infectious diseases

## Abstract

**Background:**

Digital health is a dynamic field that has been generating a large number of tools; many of these tools do not have the level of maturity required to function in a sustainable model. It is in this context that the concept of global goods maturity is gaining importance. Digital Square developed a global good maturity model (GGMM) for digital health tools, which engages the digital health community to identify areas of investment for global goods. The Surveillance Outbreak Response Management and Analysis System (SORMAS) is an open-source mobile and web application software that we developed to enable health workers to notify health departments about new cases of epidemic-prone diseases, detect outbreaks, and simultaneously manage outbreak response.

**Objective:**

The objective of this study was to evaluate the maturity of SORMAS using Digital Square’s GGMM and to describe the applicability of the GGMM on the use case of SORMAS and identify opportunities for system improvements.

**Methods:**

We evaluated SORMAS using the GGMM version 1.0 indicators to measure its development. SORMAS was scored based on all the GGMM indicator scores. We described how we used the GGMM to guide the development of SORMAS during the study period. GGMM contains 15 subindicators grouped into the following core indicators: (1) global utility, (2) community support, and (3) software maturity.

**Results:**

The assessment of SORMAS through the GGMM from November 2017 to October 2019 resulted in full completion of all subscores (10/30, (33%) in 2017; 21/30, (70%) in 2018; and 30/30, (100%) in 2019). SORMAS reached the full score of the GGMM for digital health software tools by accomplishing all 10 points for each of the 3 indicators on global utility, community support, and software maturity.

**Conclusions:**

To our knowledge, SORMAS is the first electronic health tool for disease surveillance, and also the first outbreak response management tool, that has achieved a 100% score. Although some conceptual changes would allow for further improvements to the system, the GGMM already has a robust supportive effect on developing software toward global goods maturity.

## Introduction

### Overview

Digital health is a dynamic field with a rapidly growing number of initiatives and tools, many of which operate in certain geographic areas and for a limited period [1]. This lack of sustainability may be due to a variety of reasons, including the lack of integration in standard frameworks, lack of diverse and continuous donor support, lack of serving the objectives, and limited generalizability of its application among others [2-4]. In the field of public health, funding for such initiatives relies mostly on scarce public resources. Many such parallel initiatives do not seem to be mature enough to survive their pilot phase, which makes it particularly unfortunate. It is in this context that the concept of global goods maturity is gaining importance [5,6].

### Global Goods

The concept of global goods stems from guidelines influencing health policies to support technologies that are meant to assist government agencies and policy makers in launching, scaling, and sustaining digital health innovations [7]. Different ministries of health convened advisory committees, including members of the digital health ecosystem and new multifaceted enterprises such as the Digital Impact Alliance and Digital Square [7]. These included global agencies, governments, philanthropies, funders, and academics to improve health data through shared investments in global goods and to fast-track the development and scale-up of successful digital health solutions to campaign for improvements in the facilitating environment for digital health [6,8]. To address the current lack of donor coordination, the committees created a framework to allow shared investments in specific countries and global goods. Health strengthening through enhancing country information systems would contribute to better decision making and, ultimately, better health [6]. The digital investment principles state that the funding donors within countries who are looking to prioritize their needs to improve the health of populations should align their resources around scalable, sustainable, accessible, interoperable, and evidence-based digital health global goods [6].

### Surveillance Outbreak Response Management and Analysis System

The Surveillance Outbreak Response Management and Analysis System (SORMAS) is an open-source mobile and web application software that we developed to enable health workers to notify health departments about new cases of epidemic-prone diseases, detect outbreaks, and manage outbreak response at the same time. SORMAS is a management process system that supports supervisors to validate cases and control the spread of disease. As a multifunctional software, it can be used for case surveillance, laboratory data management, contact tracing, and disease detection to prevent and manage outbreaks that may occur. SORMAS also uses a bidirectional information exchange synchronizing user requests as well as sending feedback to the different users within the existing surveillance system [9-11].

### Digital Square Global Goods Maturity Model

Digital Square, which is an initiative aimed at coordinating international efforts to develop and broadly share useful, free, and open-source digital tools, included 18 mobile health (mHealth) tools into the database of digital health software referred to as “global goods software” [12,13]. Digital Square developed a global good maturity model (GGMM) for digital health tools, which engages the digital health community to identify areas of investments for global goods [12,13]. We identified the GGMM to be a suitable concept in assessing the maturity of SORMAS for the following reasons: (1) GGMM has a particular focus on health software and on that being used in low-resource settings [12,13], (2) the objectives of GGMM matches well with the mission of SORMAS [12,13], and (3) the scope of most of the tools included in the GGMM guidebook fits that of SORMAS [12,13].

The following key concepts are prevalent throughout the study in determining the maturity of SORMAS according to the GGMM: global utility, community support, and software maturity. The objective of this study was to assess the level of global goods maturity that SORMAS has attained using the GGMM version 1.0.

## Methods

We applied the GGMM version 1.0 on SORMAS from November 2017 until October 2019 to assess the level of global goods maturity that it has attained [13]. The GGMM contains 15 subindicators grouped into the following 3 core indicators: (1) global utility, (2) community support, and (3) software maturity. Each subindicator is divided into 3 possible values from −1 for “low,” 0 for “medium,” to 1 for “high.” Each value contains a definition that is listed in Table 1. Given that the GGMM scoring values range from negative to positive values, we applied the following transformation to allow percentage values for summary scores, as shown in equation (1), where, MS=maturity score for each core indicator, Si=vector containing scores of the subindicators, and i= *1,..., 5*:

MS=5 * [MEAN (S_i_)] + 5 (1)

**Table 1:** Global good maturity model 1.0 guideline of digital intervention tools containing 15 subindicators from global utility, community support, and software maturity global indicators.

Indicator		Low	Medium	High
**Global utility**				
	Country utilization	Less than two countries or states actively use the tool for use as part of their health information system	At least four countries or states actively use the tool for use as part of their health information system, with at least 20% of total nation-wide or state-wide target users routinely using product or service as intended	At least ten countries or states actively use the tool for use as part of their health information system, with at least 30% of total nation-wide or state-wide target users routinely using product/service as intended
	Country strategy	Less than two countries or states have included the tool as part of their electronic health (eHealth) strategy or framework	At least four countries or states have included the tool as part of their eHealth strategy or framework	At least ten countries or states have included the tool as part of their eHealth strategy or framework
	Digital health interventions	The tool does not meet digital functional requirements (as defined by World Health Organization’s [WHO’s] Classification of Digital Health Interventions) without significant customization or configuration	The tool does partially meet digital functional requirements (as defined by WHO’s Classification of Digital Health Interventions) without significant customization or configuration	The tool does fully meet digital functional requirements (as defined by WHO’s Classification of Digital Health Interventions) without significant customization or configuration
	Source code accessibility	Source code not publicly available or not released under an open-source license	Source code exists on a publicly accessible repository and licensed under an open-source initiative approved license	Source code exists on a publicly accessible repository and licensed under an open-source initiative approved license. The software is structured to allow local customizations and new modules and functionality without requiring forking of main code
	Funding and revenue	At most, two revenue streams exist. Revenue streams are largely dependent on time-bound project implementations	Multiple revenue streams/funders exist across project implementations	Multiple revenue streams and funding mechanisms exist, including at least one that provides for multi-year support of core software development, documentation, and other key artifacts
**Community support**				
	Developer, contributor, and implementer community engagement	Less than 10% of the estimated total of developers, contributors and implementers are on a communication platform	Up to 20% of the estimated total of developers, contributors, or implementers, including some country representation, are engaged on a communication platform	At least 30% of estimated total developers, contributors, and implementers are engaged on a communication platform. Community leadership includes representation from countries where the tool is deployed
	Community governance	There is no community governance structure in place to direct continued development of the digital health tool	Some informal processes for community management exist to direct continued development of the digital health tool	Formal community structures (eg, leadership, technical advisory group, and community representatives) exist and are practiced with documented roles and responsibilities in a transparent fashion and are used to direct continued development of the digital health tool
	Software roadmap	No software roadmap exists, or there is no publicly accessible and routinely maintained platform for new feature requests	There is a publicly accessible and routinely maintained platform for new feature requests. A software roadmap exists describing currently planned and resourced development activities	New features and functionality are documented as part of a software roadmap as part of a release cycle. There are forums for community members to discuss new feature requests. A clear prioritization process exists and is utilized for the development of new features and functionality as part of a product backlog
	User documentation	No user documentation exists	Some user documentation exists (training manual, demo videos) but only addresses a limited subset of common functionality	A full suite of user documentation exists, including training manuals, web-based courses, tutorials, and implementation guides addressing most of the common functionality. Documentation has been released under a Creative Commons license
	Multilingual support	Limited or no support in the software for multiple languages. Multilingual documentation/user resources are practically nonexistent	Software has been internationalized to support multiple languages (though may not have been translated) for primary portions of the user interface. Some user documentation exists in more than one language	Software has been translated into multiple languages and fully supports internationalization requirements. There is an easy tool for new translations to be added. Significant parts of user and implementer documentation has been translated into at least one other language
**Software maturity**				
	Technical documentation	No substantial documentation of the software exists	Some technical documentation exists of the source code, use cases, and functional requirements	Source code is documented to the point that new adopters can customize and add new functionality with relying on significant help from one of the core developers. Online courses or tutorials are available to address common development and deployment tasks. Core business workflows and functional requirements are fully documented using use cases, user stories, or other equivalent methodology
	Software productization	No documentation available for deployment and configuration	Full documentation available for deployment and configuration. A new implementation does not require the involvement of the core development team	Software has been packaged for one or more common operating systems or platforms. Software upgrades can largely be achieved without manual intervention. Unit or integration testing is part of the release process
	Interoperability and data accessibility	Extract or import data into the system usually requires looking at source code and/or directly accessing database	Some application programming interfaces (APIs) are available for accessing and managing data. There are user-facing interfaces to export core data and metadata in the system (eg, in CSV format) for further analysis and data transfer purposes	A robust API is available for key data and metadata exchange needs for the primary business domain with functional requirements for the API having been developed in conjunction with appropriate country, regional, and global stakeholders. API endpoints exist for core data and metadata elements that adhere to standards developed by an appropriate Standards Development Organization relevant to the tools business domain. Standards-based API endpoints are used in at least four jurisdictions (eg, countries or states)
	Security	No security controls or implementation guidance is in place	Role-based authorization exists, if appropriate. Guidance on encrypting all remote access (web interface and APIs) is available to implementers	Role-based authorization exists, if appropriate. All remote access (web interface and APIs) are encrypted by default using current best practices. An independent security audit of the software has taken place within the last 12 months
	Scalability	There are no jurisdictions (eg, country and state) that manage 10% of their “entities” within the tool, and no performance and load statistics exist	There is at least one jurisdiction (eg, country and state) deployment for which 20% of all “entities” are managed within the software. There has been at least one evaluation of software performance/load testing	There is at least one jurisdictions (eg, country, state) deployment for which 30% of all “entities” are managed within the software. Performance and load testing is a part of routine releases, and results are publicly available

We put together a GGMM assessment group consisting of a public health expert from the Nigerian Centre for Disease Control, a medical epidemiologist, an international health expert, an information technology specialist and a statistician from the Helmholtz Centre for Infections Research, and 2 software engineers from Symeda, a company for developing health software. This group periodically assessed the completion of SORMAS in the 15 subindicators. Subsequently, the software roadmap and the work plan of SORMAS was reprioritized to dedicate resources to and obtain progress in those subindicators that did not reach a full score. To reduce selection bias, as members of the assessment group were part of the development and deployment of SORMAS, and to reduce the potential partiality resulting from it, we also asked 2 external experts to review the findings made by the assessment group. These external experts had contributed to the development of the GGMM and were not involved in the development of SORMAS.

## Results

In November 2017, SORMAS had migrated from a tabletop pilot version to a real-life deployed open-source software and was deployed in 8 federal states and 33 local government areas (LGAs) during the monkeypox outbreak. From September 2016 until November 2017, SORMAS was piloted in 1 state in 2 LGAs and 85 health facilities [14,15]. By February 2018, SORMAS was deployed in 3 additional federal states (71 LGAs) for meningitis outbreak. In March and April 2018, SORMAS was further deployed in 3 additional states (49 LGAs; 5 health facilities) for the Lassa fever outbreak. As of October 2019, SORMAS has been fully established for all epidemic-prone diseases in 15 federal states (including the federal capital), 287 LGAs, 37 health facilities, and approximately 700 users covering a population of 75 million. SORMAS managed multiple outbreaks simultaneously across the country. The assessment of SORMAS applying the GGMM showed that SORMAS had a 10-point score each in global utility, community support, and software maturity (see Table 2).

**Table 2:** The distribution of subindicator mean scores among the 3 core indicators (global utility, community support, and software maturity) for Surveillance Outbreak Response Management and Analysis System development from November 2017 to October 2019.

Core indicator^a^		Score			Status of SORMAS^b^
		2017	2018	2019	
**Global utility**		6	9	10	N/A^c^
	Country utilization	1	1	1	SORMAS has been fully established for all epidemic-prone diseases in 15 federal states (including the federal capital), 287 local government areas, 37 health facilities, and approximately 700 users covering 75 million population (November 2017)
	Country strategy	0	1	1	SORMAS has now been fully integrated into the revised technical guidelines of the *Integrated Disease Surveillance and Response* strategy and eHealth framework (September 2019)
	Digital health interventions	−1	1	1	SORMAS can be configured and deployed without significant customizations or configuration (July 2019)
	Source code accessibility	0	0	1	SORMAS has an open-source initiative approved license (GNU General Public License, Version 3, June 29, 2007) and is structured to allow local customizations and SORMAS is web-based and provides a relatively clear application programming interface (API) and database model. So, it is easy to build new modules and functionalities and host it on the same server (August 2019)
	Funding and revenue	1	1	1	SORMAS is has been funded by the following donors and partners since its inception; Federal Ministry of Economic Cooperation and Development and the European Union via Deutsche Gesellschaft für Internationale Zusammenarbeit, BMBF^d^, Bill and Melinda Gates Foundation, Nigerian Basic Health Care Provision Fund, US Centers for Disease Control and Prevention, Deutschen Zentrum für Infektionsforschung (DZIF). Funding sources for SORMAS increased from 1 in 2017, 3 in 2018, to 7 in 2019 (October 2019)
**Community**		3	7	10	N/A
	Developer, contributor, and implementer community engagement	−1	1	1	SORMAS currently has at least 30% of estimated total developers from Nigeria, Tanzania, Ghana, and Germany (May 2018)
	Community governance	1	1	1	In Nigeria, the steering board consists of representatives of Helmholtz Centre for Infection Research (HZI), Nigeria Centre for Disease Control, and in Ghana, the steering board includes Ghana Health Service, Ghana Community Network (GCNET), and HZI. The steering board is furthermore supported by an international external advisory board and an open-source clearance board (January 2018)
	Software roadmap	−1	0	1	New features and functionalities are documented as part of the SORMAS road map and are also part of a biweekly release cycle (May 2019)
	User documentation	0	0	1	SORMAS currently has user guides and technical documentation in which the source codes, use cases, and functional requirement exists, including training videos that are available to address everyday deployment tasks (August 2018) [16]
	Multilingual support	−1	0	1	SORMAS has a multilingual support mechanism for English and French on its platform. The language translation component in SORMAS is easy to configure by a non-information technology (IT) person and can be adapted into any language required (February 2019)
**Software**		1	5	10	N/A
	Technical documentation	0	0	1	SORMAS has full documentation for deployment and configuration, which does not require the involvement of the core development team. The SORMAS mobile app has only been packaged for the Android version operation system and not yet packaged for the iPhone operating system. The SORMAS web app has been packaged for Windows, Apple, and Linux operating systems (August 2018)
	Software productization	−1	0	1	SORMAS has automatic software upgrades without the manual intervention of the developers and also has integrated unit testing as part of the release process (August 2019)
	Interoperability and data accessibility	−1	0	1	API end points exist within SORMAS for accessing and managing data, and SORMAS has user interfaces to export core data and metadata in the system (CSV format) for further analysis and data transfer purposes (August 2017)
	Security	−1	0	1	Role-based authorization exists within SORMAS and all remote access via the web interface and APIs are encrypted by default. SORMAS has undergone an independent security audit of the software, which has taken place within the past 12 months (May 2019)
	Scalability	−1	0	1	We have deployed SORMAS in at least 30% of all entities, which are managed within the software. There has been a surge in the number of users and deployments across the country in the last year (Indicator 2, subindicators J criteria). For every SORMAS release, we evaluate the software performance and perform load testing and IT integrated testing (October 2019)
Total score, n (%)		10 (33)	21 (70)	30 (100)	N/A

^a^The global good maturity model assigned scores for each subindicator as −1 for “low,” 0 for “medium,” and 1 for “high,” and computed average values for the 5 subindicators of each core indicator using the formula in equation (1) MS=5 *[MEAN (Si)] + 5, where, MS=Maturity score for each core indicator S, i=subindicator, i=1,...,5 (vector containing score of the subindicators).

^b^SORMAS: Surveillance Outbreak Response Management and Analysis System.

^c^N/A: not applicable.

^d^BMBF: German Federal Ministry of Education and Research

## Discussion

### Principal Findings

SORMAS has reached the full score of the GGMM. The process from the decision to migrate a prototype based on a Systems, Applications, and Products proprietary technology stack to open-source software in 2016 until the accomplishment of the full score lasted 3 years [10]. There is no registry or publicly available documentation of tools that have applied the GGMM assessment except the District Health Information System 2 [17]. Of all the mHealth tools selected as global goods software listed in the GGMM guidebook, none have accomplished over 90% of the full score nor are we aware of any other tool that has obtained it [13]. Thus, it appears very likely that SORMAS may be the first and, so far, only digital tool that has reached the full score of the GGMM. SORMAS has multiple revenue streams and funding mechanisms that have been enabling progress in its development since 2016. SORMAS requires funding for software adaptations to country-specific requests, personnel for training, and supervision and maintenance of the tool.

The outline and the scoring principle of the GGMM version 1.0 was easy to apply. However, there were some limitations concerning the clarity of the definition of each of the 3 possible criteria of the subindicators. Many of those definitions contained a combination of several contextually independent items, for which the wording did not clearly distinguish between “AND” or “OR” combinations. Some definitions left room for interpretation, which may be necessary for some but also be too ambiguous in other situations. The 30 subindicators grouped into 3 core indicators all have equal weights keeping the model and its handling simple.

On the other hand, it may also not represent the difference in importance that different indicators may have. For example, it may be considered essential for global goods maturity to have repetitive external security tests implemented than multilinguarity. Once the full score has been accomplished, these differences are no longer relevant. However, as long as the total score is only partially completed, the quantitative value may be very misleading in the attempt to compare tools. A way to improve this dilemma would be to add weights to each indicator. A more natural way would be to discourage the computation of proportional completion and to categorically apply a dichotomous all-or-nothing principle, by which all requirements have been either fully fulfilled or not fulfilled [18]. Some indicators address items that may develop into two directions. For example, the number of countries using the tool may not always diminish but also increase, or an external penetration test may not be repeated as required. It may increase scaling if those subindicators that do require a regular renewal or update be explicitly highlighted to facilitate monitoring of the status. Another difficulty appeared with the subindicator for global utility, “Source Code Accessibility,” as it was not entirely clear what “new modules” and “functionality” meant in the context of this model: In contrast to modular systems, some mHealth apps are built specifically to deliver a ready-to-go solution for a specific task, and they are defined by external standards that govern how the internal processes of the end users work. In such a situation, the requirement for facilitating options for developing new modules and functionalities without prematurely forking the code may not be applicable.

For the time being, the GGMM serves as a self-assessment system. In that, the authors can confirm that in the case of SORMAS, it has dramatically guided the prioritization and acceleration of its development. It appears to be the main objective of the model. On the other hand, self-assessment also allows for a certain degree of subjectivity or bias [19]. To reduce this risk, we contacted 2 external experts who were not involved in the development and deployment of SORMAS but were instead instrumental in the development of the GGMM. Through this we aimed to reduce conflicts of interest and maximize the expertise on SORMAS as well as on the interpretation of the GGMM. We believe that inviting independent external experts in reviewing this assessment may be a model for other projects and tools to apply the GGMM assessment. We do not entirely recommend an external assessment, as practiced in many accreditation procedures, because it carries the risk of creating a business that will only but draw resources needed for the actual development of the tools.

### Conclusions

To our knowledge, SORMAS is the first electronic health tool for disease surveillance, and also the first outbreak response management tool, that has reached the full score (100%) of the GGMM. The GGMM is clear in most of its definitions and is easy to apply for self-assessment, although some indicators require more resources for completion than others. Some conceptual modifications would allow for further improvements to the system. Nevertheless, it already has a supportive effect on developing software toward global goods maturity.

## Abbreviations

GGMM: global good maturity model
LGA: local government area
mHealth: mobile health
SORMAS: Surveillance and Outbreak Response Management and Analysis System
